# FP-nets as novel deep networks inspired by vision

**DOI:** 10.1167/jov.22.1.8

**Published:** 2022-01-13

**Authors:** Philipp Grüning, Thomas Martinetz, Erhardt Barth

**Affiliations:** 1 Institute for Neuro- and Bioinformatics, University of Lübeck, Lübeck, Germany

**Keywords:** deep networks, FP-nets, hypercomplex cells, end-stopping, efficient coding, curvature, hyperselectivity

## Abstract

Feature-product networks (FP-nets) are inspired by end-stopped cortical cells with FP-units that multiply the outputs of two filters. We enhance state-of-the-art deep networks, such as the ResNet and MobileNet, with FP-units and show that the resulting FP-nets perform better on the Cifar-10 and ImageNet benchmarks. Moreover, we analyze the hyperselectivity of the FP-net model neurons and show that this property makes FP-nets less sensitive to adversarial attacks and JPEG artifacts. We then show that the learned model neurons are end-stopped to different degrees and that they provide sparse representations with an entropy that decreases with hyperselectivity.

## Introduction

For machine learning to work, one needs appropriate biases to constrain the solution for the problem at hand. Deep convolutional neural networks (CNNs), for example, are successful due to two constraints that specialize them relative to more general networks such as the multilayer perceptron (MLP): sparse connections and shared weights. It is well known that biases cannot be learned from the data or derived by logical deduction (Watanabe, 1985). In computer vision, appropriate biases can be obtained, as in the case of the CNNs, by studying biological vision (LeCun et al., 2015; Majaj & Pelli, 2018). Besides inspiring the use of localized (oriented) filters (the two CNN biases above) followed by a pointwise nonlinearity, biological vision can provide additional insight, an issue that currently receives somewhat limited attention in the deep-learning community (Majaj & Pelli, 2018; Paiton et al., 2020).

We here focus on the principle of efficient coding (Barlow, 1961; Simoncelli & Olshausen, 2001) and the related neural phenomenon of end-stopping (Hubel & Wiesel, 1965). Statistical analysis shows that oriented linear filters reduce the entropy of natural images by encoding oriented straight patterns (one-dimensional [1*D*] regions) such as vertical and horizontal edges (Zetzsche et al., 1993). In cortical area V2, however, the majority of cells are end-stopped to different degrees (Hubel & Wiesel, 1965). End-stopped cells are thought to detect two-dimensional (2*D*) regions such as junctions and corners. Since 2*D* regions are unique and sparse in natural images (Barth & Watson, 2000; Mota & Barth, 2000; Zetzsche et al., 1993), they represent images efficiently, that is, with a high degree of sparseness and minimal information loss. A standard way of modeling end-stopped cells is to multiply outputs of orientation-selective cells, resulting in an AND-combination of simple-cell outputs (Zetzsche & Barth, 1990). For example, a corner can be detected by the logical combination of “horizontal edge AND vertical edge.” In Paiton et al. (2020), the authors argue convincingly that principles adopted from vision should be beneficial for deep networks and that the exploitation of multiplicative interactions between neurons has not been sufficiently explored in this specific context. There is, nevertheless, a vast literature on sigma-pi networks in general (e.g., Mel & Koch, 1990; Rumelhart et al., 1986), which is not surprising since such networks define a large class of possible systems.

It has been shown that end-stopping can emerge from the principle of predictive coding based on recursive connections (Rao & Ballard, 1999); the latter has also been observed in Barth and Zetzsche (1998). Note that in Rao and Ballard (1999), end-stopping emerges based on unsupervised learning with natural images and, in our case, on task-driven supervised learning in a natural vision task.

Feature-product networks (FP-nets) implement a network architecture that contains explicit multiplications of the feature maps obtained with pairs of linear filters. The main feature of these networks is that they learn the appropriate filter pairs to be multiplied based on the task at hand. An early FP-net architecture has been presented as a preprint (Grüning et al., 2020b), and it has been shown in Grüning et al. (Grüning & Barth, 2021) that a similar network can predict subjective image quality well. Of course, we do not assume that neurons would compute ideal multiplications; the AND terms could be created in alternative ways, for example, by using logarithms (Grüning et al., 2020b) or the minimum operation (Grüning & Barth, 2021a) instead of multiplications. AND terms could also be generated by traditional CNNs with linear filters followed by simple ReLU nonlinearities (Barth & Zetzsche, 1998), but this would require larger networks and would be limited in terms of the possible tuning properties of the resulting nonlinear functions (see also Paiton et al., 2020, regarding the limits of pointwise nonlinearities). Here, we present a novel FP-net architecture that is closer to vision models than the ones introduced previously in Grüning and Barth (2021b) and Grüning et al. (2020b). We first demonstrate its performance and then analyze the learned units by relating them to biological vision.

Regarding the use of multiplicative terms in CNNs, Zoumpourlis et al. (2017) have shown that quadratic forms added to the first layer of a CNN can improve generalization. An FP-net can be interpreted as a special case of a network with an additional second-order Volterra kernel, but it has much fewer parameters. However, CNNs are also special cases of MLPs and, as we have argued above, the challenge is to find the right biases that can take us from the general to the more special case. For more comprehensive overviews on how FP-nets relate to various deep-network architectures, especially to bilinear CNNs (Li et al., 2017), see Grüning et al. (2020a) and Grüning and Barth (2021b). In addition, we would like to mention recent work of Chrysos et al. (2020), which illustrates that the Hadamard product of layers in deep network and the resulting higher-order polynomial representation can improve classification performance. Finally, in recurrent networks, multiplications are used to implement useful gating mechanisms (Collins et al., 2016).

## FP-nets as competitive deep networks

**Figure 1.**
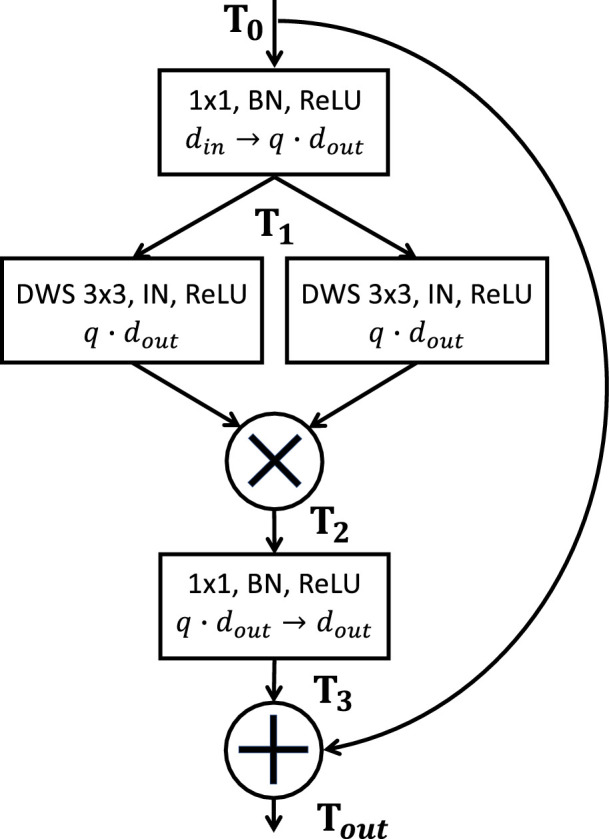
The structure of an FP-block is illustrated with rectangles and circles for the various operations applied to the input tensor $T_{0}$ gradually transforming it into $T_{out}$. The first row within each rectangle denotes which operations are applied in sequence. In the second row, the number of feature maps is given and $d_{in}\to qd_{out}$ indicates that the input number of feature maps $d_{in}$ changes to $qd_{out}$. The arrows in the figure indicate the inputs to the different operations and are labeled with the tensors defined in the equations (see text). Note that $T_{1}$ is input to two different depthwise-separable 3×3 convolutions (DWS, middle rectangles) that are learned. Convolutions are followed by instance normalization (IN) and ReLU nonlinearity, resulting in two different tensors. $T_{2}$ is the result of element-wise multiplication of these two tensors (see Equation 2). A second linear combination, depicted by the bottom rectangle, yields $T_{3}$. For the final output $T_{out}$, a residual connection adds the input tensor $T_{0}$ to $T_{3}$ (see Equation 5).

With FP-nets, we denote a deep-network architecture that contains one or several FP-blocks. Each *block* of a deep network implements a sequence of layers and operations that transforms an input tensor $T_{0}\in R^{h\times w\times d_{in}}$ to an output tensor $T_{out}\in R^{\frac{h}{s}\times \frac{w}{s}\times d_{out}}$. A tensor consists of a number (e.g., $d_{in}$, $d_{out}$) of feature maps, each with spatial width w and height h that may be altered by a factor s. The typical input tensor for a CNN is an image, the three color channels being the feature maps. The sequence of operations in an FP-block is shown in Figure 1 and consists of three steps: (a) a first linear combination, (b) the feature product, (c) a second linear combination. In the first step, the $d_{in}$ feature maps of an input tensor $T_{0}$ are linearly combined, followed by a ReLU, to yield the tensor $T_{1}$ with $qd_{out}$ feature maps:

(1) $$\begin{array}{ccc} & & T_{1}\left[i,j,m\right]=ReLU\left(\sum_{n=1}^{d_{in}}w_{m}^{n}T_{0}\left[i,j,n\right]\right);\\ & & m=1,...,qd_{out}. \end{array}$$

$T_{1}\left[i,j,m\right]$ is the value of $T_{1}$ at pixel position (i,j) and feature map m; $w_{m}^{n}$ are learned weights and q is an expansion factor that controls the block size. By $T_{1}^{m}\in R^{h\times w}$, we denote the mth feature map of $T_{1}$. The second step is the computation of feature products, the centerpiece of the FP-block. Each feature map $T_{1}^{m};m=1,...,qd_{out}$, is convolved with two learned filters $V^{m}$ and $G^{m}\in R^{k\times k}$. Filtering is followed by instance normalization (IN) (Ulyanov et al., 2016) and ReLU nonlinearity yielding two new feature maps. Subsequently, the product of the two filter outputs is computed. For any particular image patch $X\in R^{k\times k}$, with the center pixel being (i,j), of a particular feature map $T_{1}^{m}$, the filter operation for the vectorized image patch $x=vect\left(X\right)\in R^{k^{2}}$ is the scalar product of the image patch with the vectorized filters $v=vect(V^{m})$ and $g=vect(G^{m})$:

(2) $$\begin{array}{ccc} & & \;\;\;\;\;\;\;\;\;\;\;\;\;\;\;T_{2}\left[i,j,m\right]\;=\;\frac{1}{\sigma_{v}\sigma_{g}}ReLU\left(x^{T}v\;-\;\mu_{v}\right)ReLU\left(g^{T}x\;-\;\mu_{g}\right).\;\; \end{array}$$

$T_{2}\in R^{\frac{w}{s}\times \frac{h}{s}\times qd_{out}}$ is the resulting tensor and s the stride of the filter operation. If s is greater than 1, $T_{2}$'s width and height are subsampled. μ and σ are the mean value and standard deviation of $T_{1}^{m}$ after convolution with either $V^{m}$ or $G^{m}$:

(3) $$\mu_{v}=\frac{s^{2}}{hw}\sum_{i,j}\left(T_{1}^{m}*V^{m}\right)\left[i,j\right],$$

(4) $$\sigma_{v}=\frac{s^{2}}{hw}\sum_{i,j}{\left(T_{1}^{m}*V^{m}-\mu_{v}\right)}^{2}\left[i,j\right],$$

with $\left(T_{1}^{m}*V\right)\left[i,j\right]$ being the (i,j)th pixel of the filter result. In the third step, a second linear combination transforms $T_{2}\in R^{\frac{h}{s}\times \frac{w}{s}\times qd_{out}}$ into $T_{3}\in R^{\frac{h}{s}\times \frac{w}{s}\times d_{out}}$. To comply with the baseline architectures ResNet and MobileNet, a residual connection defines the final output as:

(5) $$T_{out}=T_{0}+T_{3}.$$

**Figure 2.**
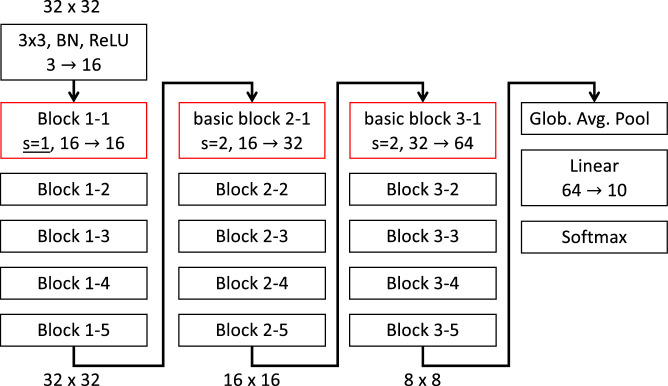
Architecture of the ResNet-32 used on Cifar-10: The network contains three stacks with five blocks each. Each block contains several layers such as convolution layers with a kernel of size 3×3 pixels, batch normalization (BN) layers, and ReLU and Softmax nonlinearities. Convolution layers with a stride s larger than 1 subsample the input, for example, from 32×32 pixels to 16×16 or 8×8 pixels. The number of feature maps can change within a block; for example, 16→32 indicates an increase from 16 to 32 feature maps. The FP-net has the same baseline architecture, but each first block in a stack (colored in red) is replaced with an FP-block.

Using the above FP-block, we designed four different FP-nets based on different baseline architectures: an FP-net based on (a) the original ResNet, and (b) the PyrBlockNet trained on Cifar-10, (c) a ResNet-50, and (d) a MobileNet-V2 both trained on ImageNet. A *stack* is a larger segment of the network, consisting of several *blocks*. Except for the first stack that may have a stride of 1, each new stack starts with a block with a stride of 2 that reduces the size of each feature map. Within a stack, all blocks operate on feature maps of the same size. Different network architectures may have different numbers and types of blocks. In our case, basic blocks, pyramid blocks, bottleneck blocks, and inverted residual blocks define the ResNet-Cifar, PyrBlockNet, ResNet-50, and MobileNet-V2 architecture, respectively. The block is the core module of an architecture and contains several *layers*. Layers are the smallest network building units such as convolution layers and max-pooling layers. Figure 2 shows an example of a ResNet-Cifar architecture that has three stacks with five blocks each. Each first block of the second and third stacks contains a convolution layer with stride s=2 that downsamples the input. The two other architectures that we used are similar: The ResNet-50 has four stacks with varying numbers of bottleneck blocks. The MobileNet-V2 has six stacks consisting of inverted-residual blocks.

We transform the four baseline architectures defined above into FP-nets using a simple design rule: Substitute each stack's first block with an FP-block. The input and output dimensions of the block are kept equal; only the internal operations differ.

We developed this design rule to improve upon already well-established architectures, making FP-nets practical since only a few changes need to be done to create an FP-net. To be compatible with state-of-the-art architectures, the FP-block has a structure similar to the MobileNet-V2 block (Sandler et al., 2018). We found that combinations of convolution blocks and FP-blocks work best and that larger kernel sizes do not improve performance. One way to view a stack is that it constitutes a visual processing chain for a specific image scale. One would expect end-stopping to be more useful at the beginning of this chain. Thus, we replaced the first block of each stack. Note, however, that later stacks, for example, the second and third stack in the Cifar-10 networks, already work with highly processed inputs coming from the previous stacks. Therefore, one would expect that there is a lower necessity of extracting 2*D* regions in later stacks. Indeed, we will show, when analyzing the γ values of FP-blocks, that highly selective neurons are more common in earlier stacks.

We train and test several FP-nets on the two well-known benchmarks Cifar-10 (Krizhevsky et al., 2021) and ImageNet (Deng et al., 2009).

Due to the moderate size of the data set, Cifar-10 is often used to evaluate the potential of new architectures and designs. For our experiments on this data set, we used ResNets (He et al., 2016) as baseline; see Figure 2 for an example. These networks have three stacks, each consisting of N blocks. We evaluated two types of the ResNet-20, ResNet-32, ResNet-44, and ResNet-56, with N=3, 5, 7, and 9 blocks, respectively (the numbers after the names indicate the number of convolution or linear layers). Since the first publication of the ResNet architecture, several additional blocks were proposed; see Han et al. (2017) for an overview. As two baselines on Cifar-10, we used the original ResNet and a variant using the pyramid block that we denote PyrBlockNet. For both variants, we created FP-nets by replacing baseline blocks with FP-blocks according to our design rule. We used the same number of blocks, but note that an FP-block contains one additional convolution layer in each block. The FP-net-23, FP-net-35, FP-net-47, and FP-net-59 are based on the PyrBlockNet: Each stack's first block is an FP-block, and all other blocks are pyramid blocks. Analogously, *FP-net (basic)* denotes an FP-net based on the original ResNet: Each stack's first block is an FP-block, and the remaining blocks are basic blocks.

Next, we evaluated the performance of FP-nets with the larger ImageNet data set that contains over 1.2 million training examples and 50,000 validation examples (we tested on the publicly available validation set). With an input size of at least 224×224 pixels and 1,000 classes, ImageNet poses a greater challenge than Cifar-10. We compared the ResNet-50 to two FP-net-50: one smaller net with an expansion factor q=0.8 and a slightly larger network with q=1. In both cases, for each of the four stacks of the ResNet-50, the first block was replaced by an FP-block to obtain the FP-net-50. Note that, if not explicitly mentioned, the term FP-net-50 refers to the q=1 variant.

To further validate our approach, we evaluated an FP-net based on the popular MobileNet-V2 architecture. As with the ResNet, we replaced the first block of each stack with an FP-block, using q=3.

**Figure 3.**
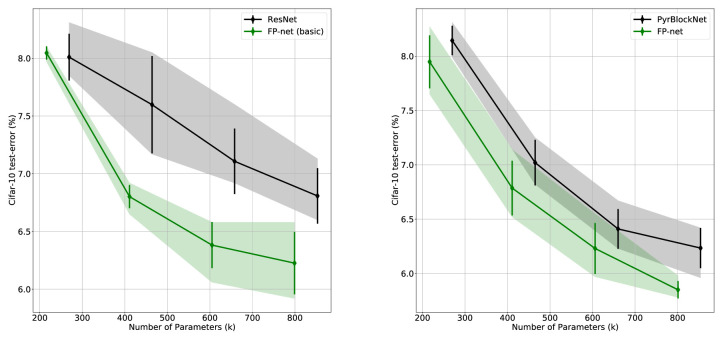
The *y*-axis displays the best test score on Cifar-10 averaged over five runs, and the bars indicate the standard deviations. The transparent area indicates the range from the minimum to the maximum. Each diamond represents one network having a specific number of parameters (in thousands) denoted on the *x*-axis. On the left, the black solid line shows the baseline ResNet results with 20, 33, 44, and 56 layers, and the green solid line the results for the corresponding FP-nets (basic). On the right, the black solid line shows the baseline PyrBlockNet and the green solid line the results for the FP-nets. Substituting each stack's first block with an FP-block yielded, in all but one case, a significantly better performance with a reduced number of parameters.

The results of the Cifar-10 experiments are shown in Figure 3: The left side compares the original ResNet to the FP-net (basic), and the right side compares the PyrBlockNet to the FP-net. Each point of the two curves shows the best possible test error occurring over all training epochs averaged over five runs and for one particular network (i.e., one particular number of blocks). The black line shows the baseline network, the green line the resulting FP-net when substituting the first blocks of the baseline's stacks. The x-axis displays the number of parameters, a number that increases with the number of blocks. Note, however, that the inclusion of FP-blocks reduces the number of parameters. Overall, the FP-nets are more compact and perform better with a lower test error and only a small overlap in the standard deviations.

**Figure 4.**
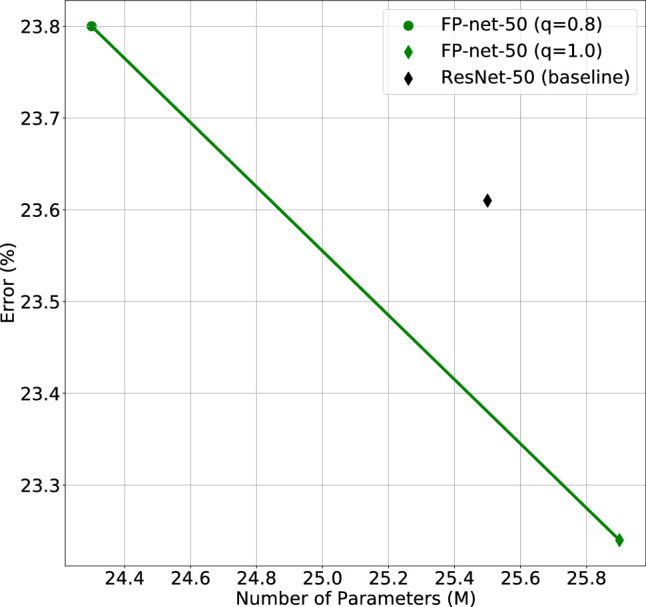
Number of parameters vs. ImageNet validation error for the ResNet-50 (black diamond) and two FP-nets (green dots) with different expansion factors q.

Table 1 shows the results on ImageNet. Note that the FP-net (q=1) performs better than the baseline ResNet-50, and the validation error is reduced by almost 0.4. When considering the already compact MobileNet architecture, the FP-net performs better than the MobileNet with an error decreased by 0.2. We trained the MobileNet-V2 baseline network ourselves to obtain its validation error. For the ResNet-50, we report the value from the Tensorpack repository (Wu, 2016). The performance depending on the number of parameters for the ResNet and FP-variants is illustrated in Figure 4.

**Table 1.** ImageNet validation errors for different FP-nets and baselines: We transformed two baseline network architectures, the ResNet-50, and the MobileNet-V2, into FP-nets, here denoted as FP-net-50 and FP-MobileNet. The transformations are done by substituting specific blocks of the baseline networks with an FP-block (see text). Additionally, by choosing different expansion factors q, we created one FP-net that is smaller than the baseline (q=0.8) and one larger network (q=1). Note that FP-nets perform better than the baseline models if there is only a slight increase in the number of parameters (shown in millions).

Model	No. of parameters (*M*)	Error
ResNet-50 (baseline)	25.6	23.61
FP-net-50 (q=0.8)	24.3	23.80
FP-net-50 (q=1)	26.0	23.24
MobileNet-V2 (baseline)	3.5	28.71
FP-MobileNet	3.5	28.53

## FP-nets and visual coding

### Hyperselectivity of FP-units

Vilankar and Field (2017) used the term *hyperselectivity* to quantify how strongly a neuron is tuned to its optimal stimulus, that is, how quickly the response drops when the optimal stimulus changes. In the context of deep learning, hyperselectivity is relevant because it can increase robustness, for example, robustness against adversarial attacks (Paiton et al., 2020). One way to quantify hyperselectivity is to measure the *curvature* of *iso-response contours*. Given an n-dimensional input to a function f, an (n-1)-dimensional surface may exist such that for all points s on the surface, the output f(s) is a constant. As n can be a high dimension, 2*D* projections are used to analyze such iso-surfaces, which in two dimensions become iso-response contours s=ϕ(t),t∈R.

**Figure 5.**
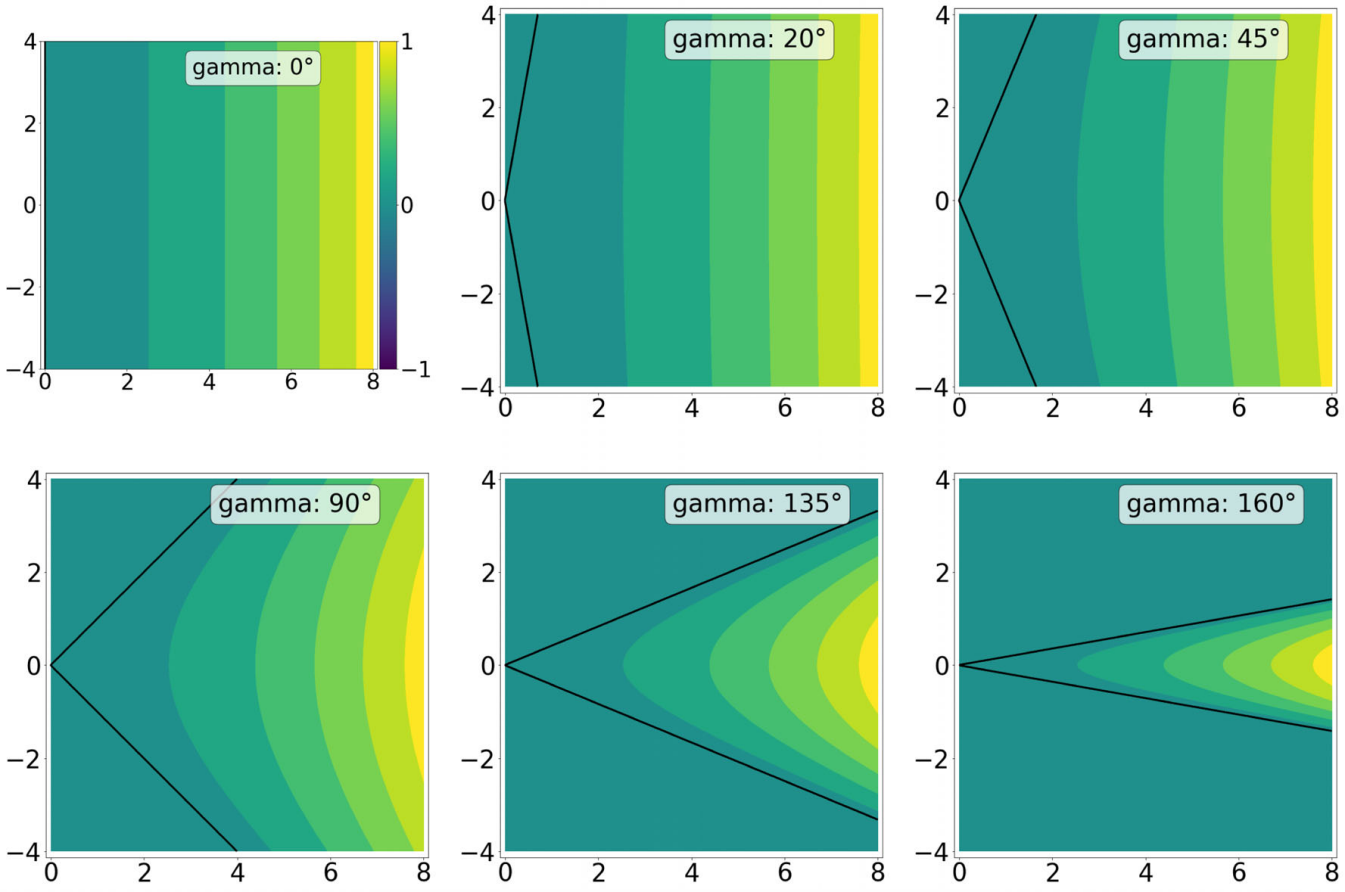
Iso-response contour plots for different values of the angle γ. Each plot shows values that were determined by using Equation 23; furthermore, normalization and quantization to six bins were applied. The horizontal axis points in the direction of the optimal stimulus and is indexed by the y value in Equation 23. The vertical axis is orthogonal to the optimal stimulus and indexed by x. The black lines indicate the zero contour.

**Figure 6.**
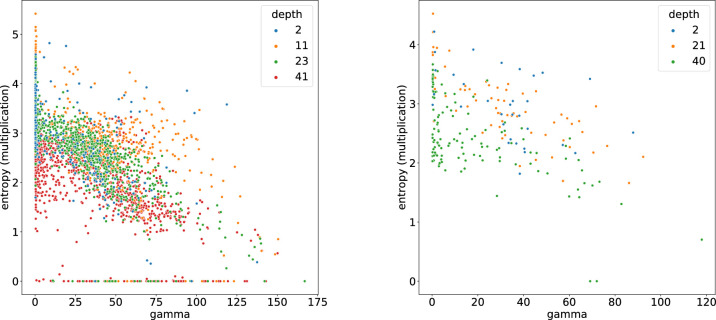
Scatterplots of entropy over hyperselectivity (indicated by the angle γ). Each dot corresponds to an FP-neuron. The color codes indicate the position of each neuron in the network (i.e., the number of convolution layers). The entropy of a particular FP-neuron's feature map is estimated as described in the Appendix and plotted against γ=∡(v,g). The left panel shows results for the FP-net-50 trained on ImageNet after 2, 11, 23, and 41 convolution layers, and the right panel shows results for the FP-net-59 trained on Cifar-10 after 2, 21, and 40 convolution layers. Note the correlation between entropy and γ. Hyperselectivity is directly linked to γ, as illustrated in Figure 5.

The typical linear-nonlinear (LN) model neuron used in CNNs is a function $f_{LN}\left(x\right)$ that involves a linear projection on a weight vector $w\in R^{n}$ followed by a pointwise nonlinearity ρ(x). To analyze the iso-response contour of such a neuron, one first projects the input on w, the axis corresponding to the optimal stimulus $x_{opt}$. To find a second axis, one searches for a vector orthogonal to $x_{opt}$, for example, by picking n random values and using the Gram–Schmidt process (see Equation 16) to transform the random vector to one that is orthogonal to $x_{\mathrm{opt}}$. When looking at the output of an LN-neuron for $x_{\mathrm{opt}}$ perturbed by any orthogonal vector z with $x_{\mathrm{opt}}^{T}z=w^{T}z=0$, the iso-response contour is always a straight line parallel to z, because $f_{LN}\left(x_{opt}+z\right)=\rho \left(w^{T}\left(x_{opt}+z\right)\right)=\rho \left(w^{T}x_{opt}\right)=f_{LN}\left(x_{opt}\right)$. Thus, for LN-neurons, the iso-response contours have zero curvature. For hyperselective neurons ($f_{HS}\left(x\right)$), there exist vectors z that are orthogonal to $x_{opt}$ and decrease the neuron's optimal response such that $f_{HS}\left(x_{opt}+z\right)<f_{HS}\left(x_{opt}\right)$. In this case, the exo-origin iso-response contour bends away from the origin of the basis defined by $x_{opt}$ and z. A higher curvature of this bend indicates a more significant activation dropoff in regions that are different from the optimal stimulus (i.e., a greater hyperselectivity). One way to quantify the curvature is to use the coefficient of the quadratic term obtained by fitting a second-order polynomial to the iso-response contour. FP-nets contain FP-blocks that consist of FP-units, or *FP-neurons*, which yield the feature-product output for a pixel (i,j) in a feature map m as defined by Equation 2. As shown in the Appendix, FP-neurons exhibit curved exo-origin iso-response contours with a curvature that depends on the angle γ=∡(v,g). Iso-response contours are shown in Figure 5 for different values of γ. Note that curvature, and thus hyperselectivity, increases with γ. Accordingly, a large γ leads to a lower entropy of the resulting feature maps; see Figure 6.

### Entropy and degree of end-stopping

To further support the view that FP-neurons are hyperselective depending on γ, we analyzed the entropy of the feature maps generated by different FP-neurons. The results in Figure 6 show that the learned filters tend to have a γ larger than zero, that is, the majority of FP-neurons are hyperselective and that a high γ-value leads to a lower entropy. Details of how the entropy is computed are given in the Appendix.

**Figure 7.**
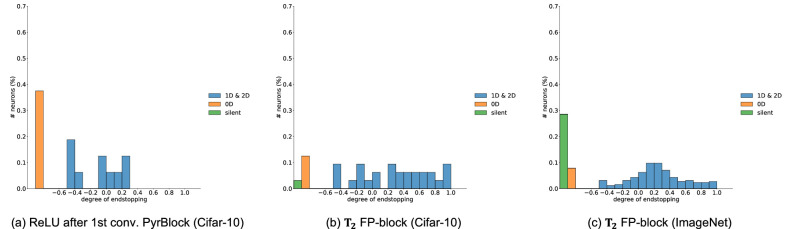
Distribution of neurons plotted over the degree of end-stopping. Distributions are shown for the first block of the first stack for different models. The left image shows the activation of the first convolution, after batch normalization and ReLU, of a pyramid block in a PyrResNet (nine blocks per stack). Middle: the FP-neuron ($T_{2}$) of an FP-block for an FP-net trained on Cifar-10 (nine blocks per stack). Right: $T_{2}$ of an FP-block for the FP-net-50 trained on ImageNet. Blue bars show normalized histograms for the ratio $1-\frac{1D}{2D}$ that quantifies the relation between responses to straight edges (1*D*) and corners (2*D*); see Appendix. Neurons that respond to 0*D* regions (the center of a square) are excluded from the blue histogram and shown separately as orange bars. Neurons that do not respond at all (0D, 1D, and 2*D* responses are all zero) are also excluded from the blue histogram and are shown as green bars.

In order to analyze the end-stopping behavior of the model neurons that are learned in the FP-nets trained on Cifar-10 and ImageNet, we needed to quantify the degree of end-stopping. In order to relate to physiological measurements, we started by analyzing the response of FP-neurons to straight lines and line ends, but this turned out to be problematic because the FP-nets use small 3×3 filters and subsample the input. To keep the analogy, but with a more robust measure, we used a square as input and quantified the average responses to the uniform zero-dimensional (0*D*) regions, the straight 1*D* edges, and the 2*D* corners. The degree of end-stopping is then defined by the relation between 1*D* and 2*D* responses. In order to account for ON/OFF- type responses, we used both a bright and a dark square. The results are shown in Figure 7, and the details of the algorithm are given in the Appendix.

Note that, as the real neurons in cortical areas V1 and V2, the model neurons in the FP-net are end-stopped to different degrees. Thus, end-stopping seems to be beneficial for both the ImageNet and Cifar-10 tasks, since the emergence of end-stopping is here driven by the classification error. As expected, the multiplication in the FP-block shifts the distribution toward a higher degree of end-stopping. However, the network could have learned filter pairs that do not lead to end-stopped FP-neurons. The bias that we introduce (i.e., the multiplication) just makes it easier for the network to learn end-stopped representations.

**Figure 8.**
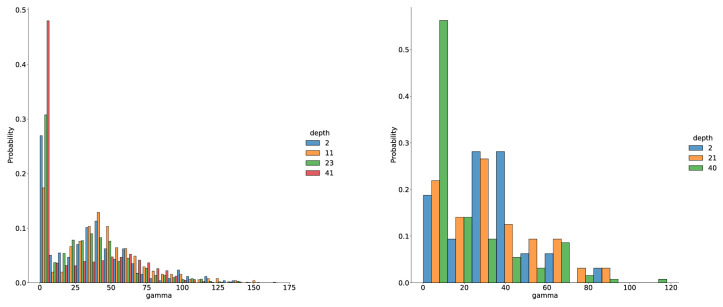
Distribution of FP-neurons ($T_{2}$) as a function of hyperselectivity (indicated by the angle γ=∡(v,g)) and for different positions in the network. Note that the majority of neurons are hyperselective to different degrees and that hyperselectivity is reduced later in the network. The left panel shows results for the FP-net-50 trained on ImageNet after 2, 11, 23, and 41 convolution layers. The right panel shows results for the FP-net-59 trained on Cifar-10 after 2, 21, and 40 convolution layers.

The angle distributions in Figure 8 show that indeed linear FP-neurons are learned as well since more than 15% of FP-neurons have a γ-value near zero. With increasing network depth, the number of linear FP-neurons increases, indicating that hyperselectivity and especially end-stopping are more frequent in earlier stages of the visual processing chain.

### FP-neurons are more robust against adversarial attacks

**Figure 9.**
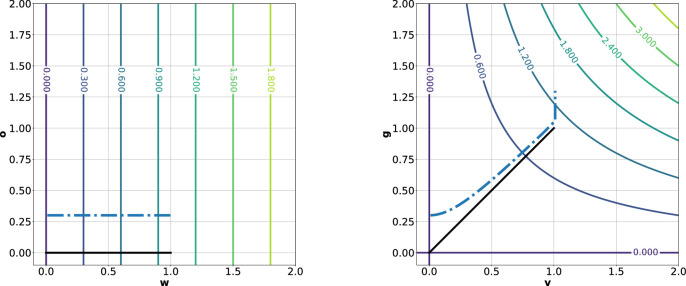
Iso-response contours and iteration path of CIGA for (left) an LN-neuron and (right) an FP-neuron: The LN-neuron's weight vector is $w={\left(1,0\right)}^{T}$ and $o={\left(0,1\right)}^{T}$ is an orthogonal vector. The FP-neuron's filter-pair is $v={\left(1,0\right)}^{T}$ and $g={\left(0,1\right)}^{T}$. The black lines point in the directions of the respective optimal stimulus. The blue dashed line shows the iteration path of the CIGA (see Equation 6). All other colored solid lines show iso-response contours; the number on each line shows the function value of the contour. For each neuron, CIGA aims to find a perturbation η with ${∥\eta ∥}_{\infty }\leq \varepsilon =1$ that maximally increases the output f(x+η). $x={\left(0,0.3\right)}^{T}$ is the initial input to the neurons; τ=0.001 is the step size, and a total of 10,000 iterations were computed. CIGA quickly finds an optimal solution for the LN-neuron since any step along the positive gradient (parallel to the optimal stimulus, orthogonal to the iso-response contours) optimally increases the function value. For the FP-neuron, the iteration path first moves toward the optimal stimulus, then almost parallel to it, and finally, moves upward once the bound on ε is reached along the v-axis. This longer, more complex optimization path shows that CIGA is less effective for a hyperselective FP-neuron, indicating that FP-neurons are more robust against adversarial examples.

Although outperforming almost all alternative approaches on many vision tasks, CNNs are surprisingly sensitive to barely visible perturbations of the input images (Szegedy et al., 2013). An adversarial attack on a classifier function f adds a noise pattern η to an input image x so that f(x+η) does not return the correct class y=f(x). Furthermore, the attacker ensures that some p-norm of η does not exceed ε. In many cases, including this work, the infinity-norm is chosen, and the ε values are in the set {1/255,2/255,...}. Thus, for example, for ε=1/255, each 8-bit pixel value is at most altered by adding or subtracting the value 1. Goodfellow et al. (2014) argue that the main reason for the sensitivity to adversarial examples is due to the linearity of CNNs: With a high-dimensional input, one can substantially change a linear neuron's output, even with small perturbations. Consider the output of an LN-neuron for an input x with dimension n perturbed by η. We choose η to be the sign function of the weight vector multiplied with ε: η=sign(w)·ε. Thus, η roughly points in the direction of the optimal stimulus (which is also the gradient), but its infinity-norm does not exceed ε. Assuming that the mean absolute value of w is m, $f_{LN}\left(\eta \right)$ is approximately equal to εnm. Accordingly, a significant change of the LN-neuron's output can be achieved by a small ε value if the input dimension n is large, which is the case for many vision-related tasks. This gradient-ascent method can also be applied to nonlinear neurons. Within a local region, the output of almost any function f can be approximated by a linear function. To optimally increase the output, the input needs to be moved along the gradient direction. The fast gradient sign method (FGSM; Goodfellow et al., 2014) perturbs the original input image x by adding η=εsign(∇f(x)). Another approach is to define η to be the gradient times a positive step size τ followed by clipping to $\eta \in {\left[-\varepsilon ,+\varepsilon \right]}^{n}$. The clipped iterative gradient ascent (CIGA) greedily moves along the direction of the highest linear increase,

(6) $$\begin{array}{c} \begin{array}{ccc} \eta_{0} & = & 0;\tau >0\\ q_{i+1} & = & \eta_{i}+\tau \nabla f\left(x+\eta_{i}\right)\\ \eta_{i+1}^{j} & = & \min(\max\left(q_{i+1}^{j},-\varepsilon \right),\varepsilon ), \end{array}\; \end{array}$$

with $q_{i}^{j}$ being the *j*th entry of the unbounded result $q_{i}$ at the *i*th iteration step. In the following, we use CIGA in our illustrations of the principle, and in our experiments, we employ FGSM as it is a widely recognized adversarial attack method. When regarding an iso-response contour plot, one can easily spot the direction of the gradient, which is orthogonal to an iso-response contour (Paiton et al., 2020). In Figure 9 on the left, the gradient for an LN-neuron is parallel to the optimal stimulus (black line). As long as the initial input yields a nonzero gradient, each step of CIGA maximally increases the LN-neuron output. Thus, the algorithm's effectiveness is only bounded by ε but widely independent of the initial input x. For a step size larger than ε, CIGA finds the optimal solution in one step. We now investigate the effects of CIGA on a simplified version of an FP-neuron:

(7) $$F\left(x\right)=x^{T}vg^{T}x.$$

Note that in the following particular example, the input is chosen to yield nonnegative projections on v and g; thus, we can remove the ReLUs. The resulting gradient is

(8) $$\nabla_{F}\left(x\right)=\left(v^{T}x\right)g+\left(g^{T}x\right)v.$$

The effectiveness of an iteration step strongly depends on the current position. The highest possible increase would be obtained along the line defined by the optimal stimulus. In Figure 9 on the right, this is the black line. If the initial input is located on this line, any step in the gradient direction yields an optimal increase of the FP-neuron output. However, for any other position with a nonzero gradient, an unbounded iteration step would move toward the optimal stimulus line. The blue curve in Figure 9 shows the path for several iterations of CIGA: Starting above the optimal stimulus line, each step slowly converges to the optimal stimulus line, eventually moving almost parallel to it. Once the ε threshold of 1 is reached in the horizontal dimension, the (now bounded) path runs parallel to the vertical dimension to increase the neuron output further. The optimal solution is found once the ε bound is also reached in the vertical dimension. The important difference when comparing with LN-neurons is that there are numerous conditions (depending on τ, x, γ, and ε) where CIGA would need several steps to find an optimal solution. This reduced effectiveness of the gradient ascent illustrates why hyperselective neurons are more robust against adversarial attacks; for example, if ε is too small, or τ is chosen poorly, or with too few iterations, an attack might not increase the FP-neuron output by much. Note that single neurons are usually not the target of adversarial attacks; instead, the gradient is determined on the classification loss function. Still, the argument holds that hyperselective neurons are harder to activate than LN-neurons, resulting in an increased robustness.

To test this hypothesis, we created new Cifar-10 test sets $S_{\varepsilon_{i}}=\left\{FGSM\left(x,\varepsilon_{i}\right):x\in X_{C10}\right\}$ derived from the original test set $X_{C10}$. Here, we focused on the most subtle adversarial attacks: we created one test set $S_{1/255}$, where each test image was perturbed by using FGSM with ε=1/255. Results for larger ε-values are shown in the Appendix (see Table 2 and Table 3). To exclude the hypothesis that the better accuracy (with perturbations) is due to the fact that the FP-nets already generalize better, we present results where we measure the percentage of changed predictions of the classifier f.

(9) $$\begin{array}{ccc} & & Perc.\;of\;changed\;predictions(f,\Gamma ,\theta )\\ & & \;=\frac{1}{|X_{C10}|}\sum_{x\in X_{C10}}1\left(f\left(x\right)\neq f\left(\Gamma \left(x,\theta \right)\right)\right),\; \end{array}$$

**Figure 10.**
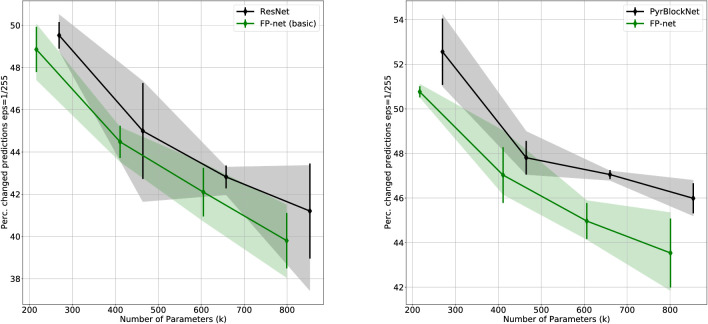
Percentage of changed predictions (see Equation 9) on the adversarial example test set $S_{1/255}$. A lower value indicates that a network is more robust against attacks created by the FGSM.

1 is the indicator function returning a 1 for a true statement and a zero otherwise. Γ is some function (here, FGSM) that perturbs the original image x based on some parameter θ. We evaluated this metric for each of the four architectures that we trained on the original Cifar-10 training set (see Section “FP-nets as competitive deep networks”); no additional adversarial training scheme was employed. As shown in Figure 10, 40% to 50% of the predictions did change. However, for both baseline models, substituting some of the LN-neurons with FP-neurons increased the robustness against FGSM attacks.

**Figure 11.**
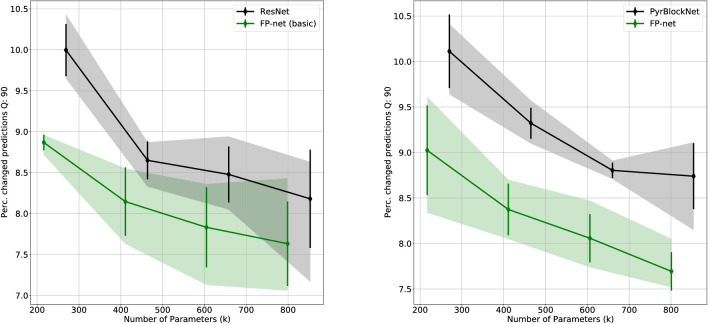
Percentage of changed predictions (see Equation 9) for the JPEG-compressed Cifar-10 test set $S_{90}$. A lower value indicates that a network is more robust against JPEG artifacts.

The results reiterate that CNN predictions can be significantly altered by deliberate and subtle attacks (we show some example images in the Appendix). Unfortunately, this lack of robustness creates problems of practical relevance beyond such attacks. For example, JPEG-compression can create artifacts that have similar effects. To evaluate robustness against JPEG artifacts, we created the Cifar-10 test sets $S_{Q_{i}}=\left\{JPEG\left(x,Q_{i}\right):x\in X_{C10}\right\}$, with JPEG(x,Q) being the JPEG-compressed version of the original image x with a quality rate Q∈{1,2,...,100}, 100 being the original image. A low quality indicates a high compression with stronger artifacts (example images are given in the Appendix). In Figure 11, we show the results for the low compression test set $S_{90}$ and further results in the Appendix (see Tables 4 and 5).

Again, using FP-neurons increased the robustness against artifacts. However, even a moderate compression alters up to 10% of the CNNs’ predictions.

### Example FP-unit

**Figure 12.**
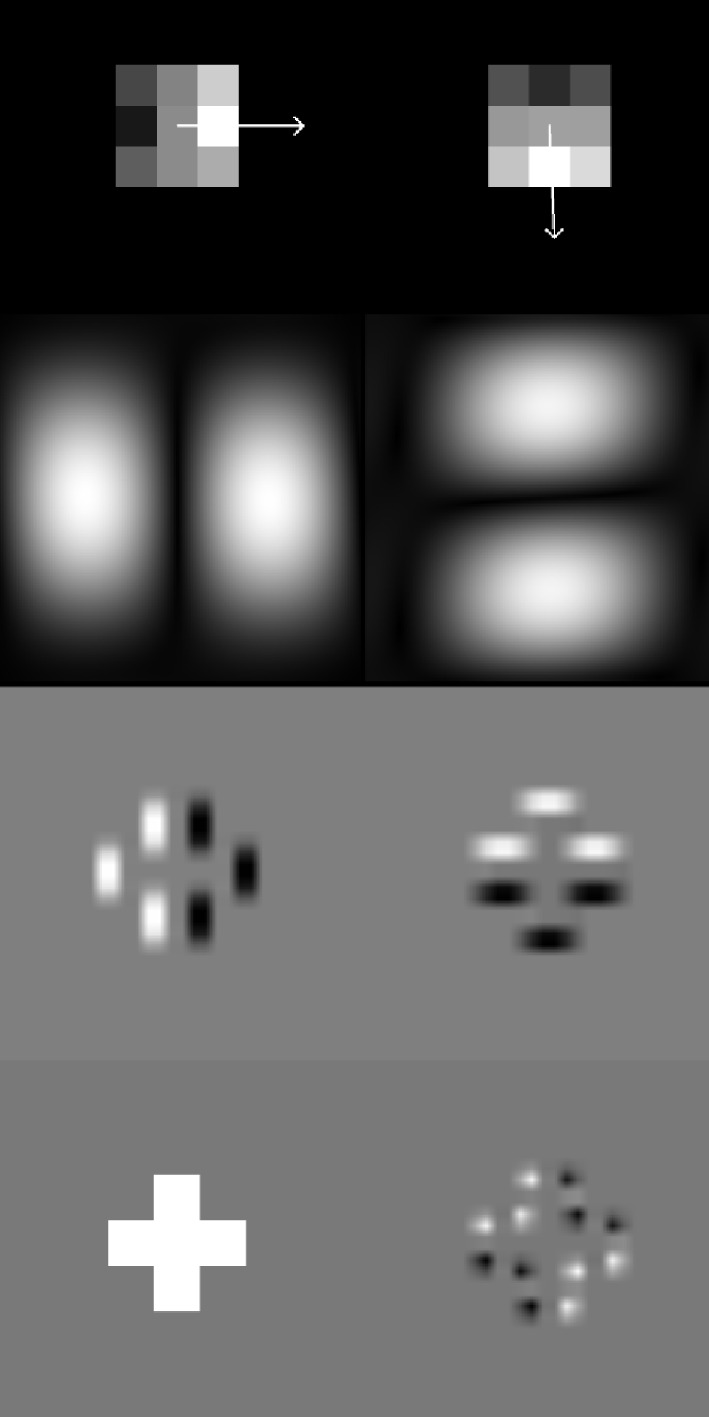
An example of learned filters pairs. The top row shows the two learned 3×3 filters (arrows indicate orientation) and the row below the corresponding Fourier spectra. The third row depicts the responses of the two filters to the image shown in the bottom left (image used as $T_{1}$ to illustrate the selectivity of the FP-unit). The bottom-right panel shows the response of the FP-unit (the product of the filter responses). Such textbook units, however, are rather rare. This particular unit has emerged in the FP-net-59 trained on Cifar-10 without instance normalization and without the ReLU in Equation 2.

As shown above, the learned FP-neurons are hyperselective and end-stopped to different degrees. However, these two properties do not fully specify an FP-neuron. When analyzing the individual FP-neurons in more detail, it is difficult to further specify them according to simple properties such as orientation or phase. Nevertheless, some FP-neurons look as if they were taken from a textbook on “how to model end-stopped neurons,” and we show one example in Figure 12.

## Discussion and conclusions

We have presented a novel FP-net architecture and have demonstrated its competitive performance. To do so, we have designed experiments with state-of-the-art deep networks and showed that we could improve their performance by substituting original blocks in the network architecture with FP-blocks that implement an explicit multiplication of feature maps. Given this simple design rule, we can expect our approach to be of practical use, since any traditional network can easily be transformed into an FP-net that will most likely perform better. We did not employ any hyperparameter tuning specific to the FP-nets but just used the hyperparameters of the original networks; one may thus expect even better performance with additional tuning. We believe that the improvement that comes with FP-nets is due to an appropriate bias, which allows the network to learn efficient representations based on units (model neurons) that are end-stopped to different degrees. The multiplications that we introduce allow for AND rather than OR combinations and thus make the resulting units more selective than linear filters with pointwise nonlinearities. Note that the key feature of FP-nets is that one learns pairs of linear filters, which are then AND combined. In case of FP-nets, the AND is implemented by multiplications. We could, however, show that logarithms (Grüning et al., 2020b) and the minimum operation (Grüning & Barth, 2021a) can also work as AND operation. We consider the improvements that bio-inspired FP-nets achieve over the baseline networks to be the main contribution of our article.

Moreover, we have analyzed the selectivity of the FP-units in an attempt to relate them to what is known about visual neurons. We could show that FP-units are indeed end-stopped to different degrees. The emergence of end-stopping in a network that learns based on only the classification error demonstrates that end-stopping is beneficial for the task of object recognition. This finding is supported by previously known mathematical results, according to which (a) 2*D* features such as corners and junctions are statistically rare in natural images, leading to sparse representations (Zetzsche et al., 1993), and (b) 2*D* features are still unique since there exists a mathematical proof that 0*D* (uniform) and 1*D* (straight) regions in images are redundant (Mota & Barth, 2000), although being statistically frequent.

Of course, the considerations above cannot be taken to imply that biological vision implements an FP-net architecture, especially as the FP-nets implement additional and typical deep-network operations such as linear recombinations that increase the entropy of the representation. In other words, much of what well-performing deep networks do is not something one would necessarily consider to be optimal.

It is known that sparse-coding units are more selective than typical CNN units, that is, than linear neurons with pointwise nonlinearities (Paiton et al., 2020), and thus less prone to certain adversarial attacks. This increased selectivity has been quantified with the curvature of the iso-response contours. We could show that the iso-response contours of the FP-units are curved, with the degree of curvature depending on the angle between the multiplied feature vectors, and that a large number of hyperselective units emerge in FP-nets trained for object recognition. Furthermore, our results show that FP-nets are indeed more robust against adversarial attacks and compression artifacts, and this is, again, due to the vision-inspired FP-units.

## Figures and Tables

**Figure 13.**
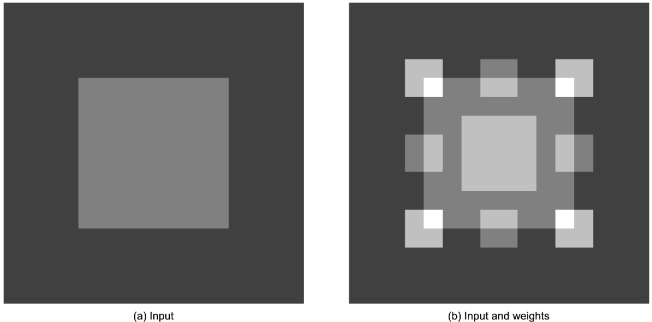
Measuring the degree of end-stopping: panel (a) shows the input image presented to the model. Subsequently, we measure the normalized activations $T_{n}^{m}$ of the feature map m of a tensor n. We determine the 0D-, 1D-, and 2D-amount via a weighted sum of the feature map and a binary region of interest image (see Equation 10). The regions are depicted in panel (b): For 0D, we measure the activation of the smaller square in the middle. For 1D, we measure the four small squares located between the corners of the input square, corresponding to the straight lines of the input signal. For 2D, we measure the four small squares located on the corners of the input square.

**Figure 14.**
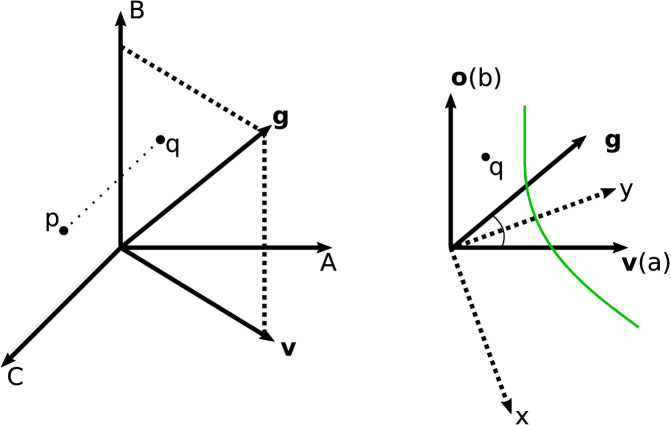
Finding the iso-response contour of the FP-neuron for a two-dimensional subspace: A high-dimensional signal space is illustrated in the left panel for just three dimensions, and the right panel displays the corresponding two-dimensional projection. To obtain the FP-neuron output for a vector $p\in R^{3}$, the scalar product of p and v is multiplied with the scalar product of p and g (see Equation 7). Note that the resulting product does not change when p is projected to q, that is, projected on the plane spanned by v and g. The coordinates of q are defined by the orthogonal axes v and o. The green curve depicts the iso-response contour: Any point on this curve results in the same activation z. The axes y and x span a basis with y pointing into the direction of the optimal stimulus (see Equation 14). The green curve is an exo-origin iso-response contour (see Equation 24) since it bends away from the direction of the optimal stimulus. The point ${\left(0,y\left(0\right)\right)}^{T}$ is the intersection of the green curve and the optimal stimulus axis y. Equation 26 shows that when moving away from this point along the x-direction, the neuron's activation decreases quadratically.

**Figure 15.**
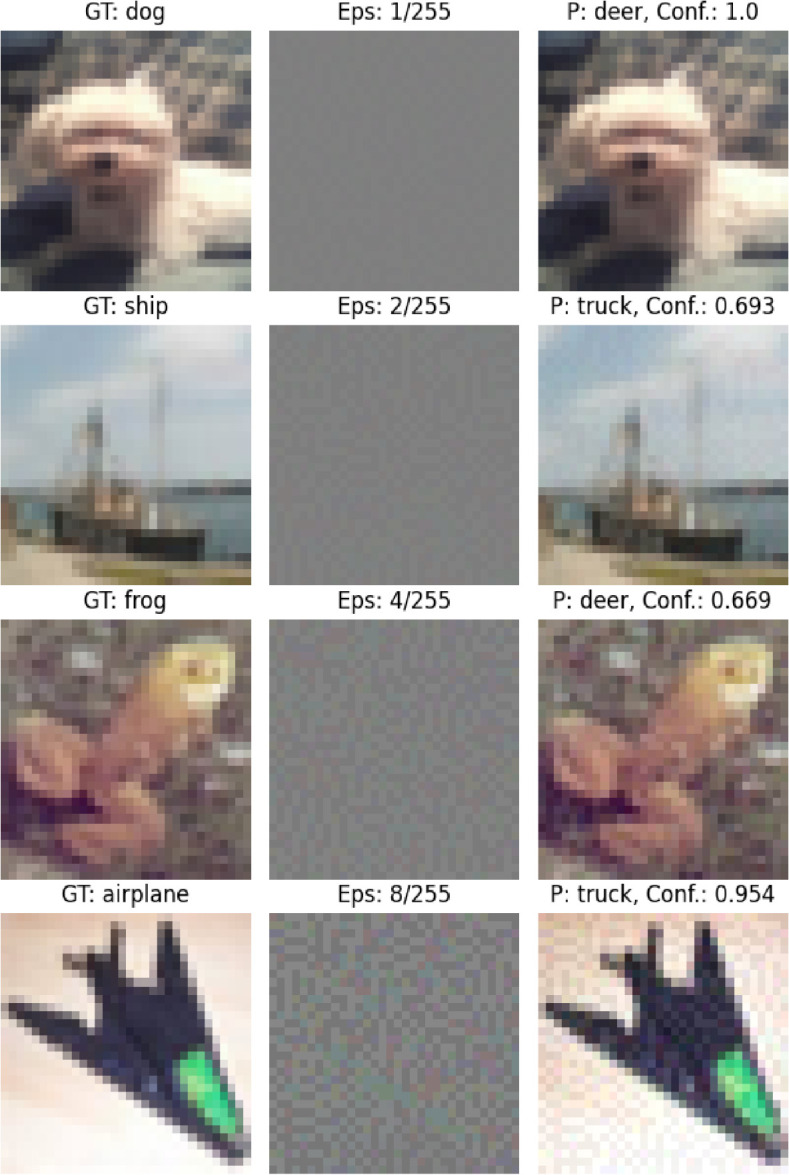
Examples of Cifar-10 adversarial attacks on the FP-net (basic) N=9 model. From left to right: original image, perturbation, and resulting adversarial example. Image titles denote the ground truth class (GT), the ε value (Eps), the predicted class (P), and the Softmax output (Conf.), that is, the confidence of the network, ranging from low =0 to high =1.

**Figure 16.**
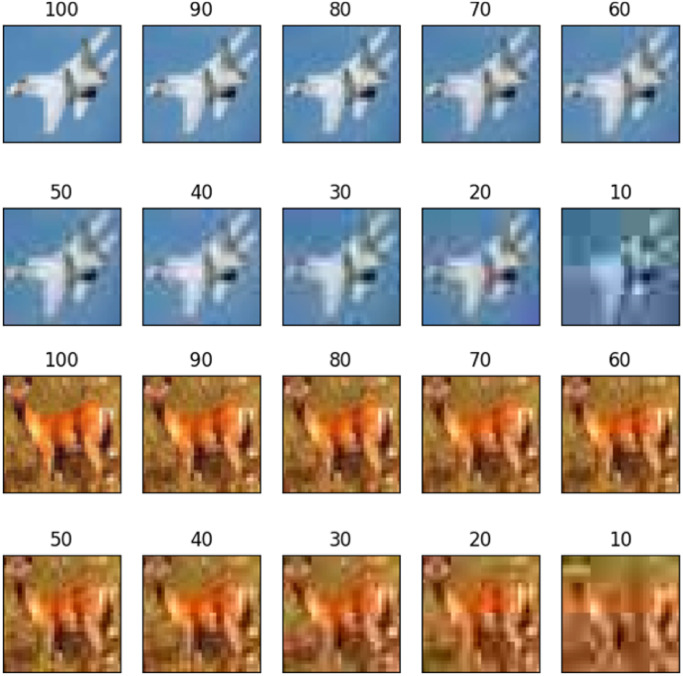
JPEG-compression examples. The number above each image denotes the image quality Q∈{1,2,...,100}. Q=100 is the original image.

## Appendix

### A.1 Details on network design and training procedure

All experiments were conducted using the PyTorch deep-learning framework (Paszke et al., 2019). Note that in all cases, for Equation 1, the output of the weighted sum has been normalized via batch normalization before applying the ReLU nonlinearity.

#### A.1.1 Residual connections

For the residual connections in Equation 5, some additional computations are needed if the dimensions of $T_{0}$ and $T_{3}$ differ. In case that $d_{out}$ is greater than $d_{in}$, zero padding is used to match the dimension of the feature maps. If $d_{in}$ is greater than $d_{out}$, an additional linear combination is learned to reduce the number of feature maps. If the FP-block's stride s is greater than 1, $T_{0}$ is subsampled by average pooling. For more implementation details regarding residual connections, see Han et al. (2017). Residual connections enable a more stable gradient flow during training, allow to better model identity functions (He et al., 2016), and enable CNNs to behave like ensembles of shallower networks (Veit et al., 2016).

#### A.1.2 Cifar-10 experiments

Cifar-10 contains 50,000 training and 10,000 test images (RGB, with height and width 32) of 10 different commonplace objects, such as airplane, bird, cat, and ship. For each FP-net and each PyrBlockNet, five experiments were conducted with five different random seeds that control the initialization of each network's random weights and the random mini-batch collection during training. The networks were trained for 200 epochs, using stochastic gradient descent (SGD), with a learning rate of 0.1 that was reduced to 0.01 and 0.001 after the 100th and 150th epoch. We used a momentum of 0.9, a weight decay of 0.0001, and a batch size of 128. For data augmentation, during training, with a probability of 50%, each input image was flipped horizontally. Subsequently, all images were padded with 4 pixels, and then a random crop of 32×32 were used. Furthermore, the RGB crop was first divided by 255 and normalized with the ImageNet mean $\mu_{imNet}\left(0.485,0.456,0.406\right)$ and standard deviation $\sigma_{imNet}\left(0.229,0.224,0.225\right)$ for the three input channels, respectively. When computing the test scores, no random cropping and no horizontal flipping were used. Each FP-block's expansion factor q was set to 2. Based on the work of Srivastave et al. (2015), the best test error was reported to better reflect the variance of the results due to different network initializations.

#### A.1.3 FP-ResNet on ImageNet

The FP-net-50 was trained for 100 epochs with randomly initialized weights using SGD on 224×224 crops with a batch size of 512. After one third, and then again after two thirds of the training time, the initial learning rate of 0.1 was decreased by a factor of 10. The weight decay was 0.0001 and the momentum 0.9. For data augmentation, we used the code from the sequential-imagenet-dataloader repository (Gray, 2017); during training, crops of various random sizes were passed to the network ranging from 8% to 100% of the original image size. The aspect ratio was chosen randomly between 3/4 and 4/3. Furthermore, different photometric distortions (e.g., random contrast changes) were applied as described in Szegedy et al. (2014) and the Tensorpack repository (Wu, 2016). When computing the test scores, each input image is first resized such that the shortest edge's length is 256. Next, the image is cropped in the center to size (224, 224), divided by 255, subtracted with 0.5, and again divided by 0.5.

#### A.1.4 MobileNet-V2 and FP-MobileNet

The FP-MobileNet was trained from scratch with SGD for 150 epochs and with a batch size of 256. The initial learning rate of 0.05 was decreased according to a cosine scheduling; see Li et al.'s repository (Li et al., 2021). The training data augmentation included random resizing and cropping, random horizontal flips, color jitters, division by the maximum value, and normalization by $\mu_{imNet}\left(0.485,0.456,0.406\right)$ and $\sigma_{imNet}\left(0.229,0.224,0.225\right)$. During testing, the input images were first resized to 255×255 and then a center crop of size 224×224 was computed. Subsequently, the crop was normalized as described above. For more information, see Fastai's repository (Howard, 2018).

### A.2 Entropy

We analyzed the entropy of all FP-neurons $T_{2}$ for the FP-ResNet-50 (ImageNet) and the FP-ResNet-59 (Cifar-10). One hundred randomly sampled images from the respective test set (in case of ImageNet, the validation set) were passed to each network. For each input, we computed the corresponding feature maps for every FP-block, one tensor $T_{2}$ for every block. We normalized each feature map $T_{2}^{m}$ from $R^{+}$ to {0,1,...,255} and computed the entropy of the pixel distribution over the 256 integer values. For the 100 input images, we obtained 100 entropy values for each feature map. We averaged these 100 values resulting in the mean entropy for each feature map (i.e., each FP-neuron).

We observed that some of the feature maps $T_{1}^{m}$ had all pixel values equal to zero (so-called dying ReLUs; Lu et al., 2019). The corresponding FP-neurons were removed from the analysis. For the FP-ResNet-50, the percentage of dying ReLUs was 23%, 0.002%, 7%, and 18% for the first, second, third, and fourth FP-blocks, respectively. For the FP-ResNet-59, only the third FP-block had 5% dying ReLUs. We tested different weight initializations or alternative nonlinearities, such as the leaky ReLU. However, although, using leaky ReLUs stopped the emergence of dying ReLUs, we only noticed a small gain in performance.

### A.3 Degree of end-stopping

To measure the degree of end-stopping, we used two input images $I_{0}$, $I_{1}$, one with a bright and one with a dark square: Pixels belonging to the square had a value of +1 or −1, respectively; all other pixels were zero. Each image was normalized to have zero mean and a standard deviation of 1. We computed the intermediate outputs $T_{2}\left(I_{0}\right)$ and squared them to obtain the activation energy. For the PyrResNet, we used the ReLU after the first convolution as intermediate output. $T_{i}\left(I_{0}\right)$ is the *i*th tensor that is computed using the image $I_{0}$ as input. We then normalized each tensor $T_{n}$ from $R^{+}$ to [0,1] by dividing it with the mean plus three times the standard deviation and clipped any values greater than 1 to make the normalization less susceptible to possible outliers. The percentage of outliers never exceeded 10%. For each feature map, we determined the values 0D, 1D, and 2D by summing the feature map pixel values (i.e., the activations) over specific regions of interest that were either homogeneous areas, straight edges, or corners in the input image:

(10) $$\psi D\left(T_{n}^{m}\right)=\sum_{i,j}T_{n}\left[i,j,m\right]W_{\psi }\left[i,j\right].$$

$W_{\psi }$ is a binary matrix used to compute the ψD value. All pixels within the region of interest are 1; the others are zero. The weighted areas are shown in Figure 13 in the right panel: The square in the middle is the region of interest for 0D. The four small squares along the straight edges of the input square measure 1D; the four small squares at the corners measure 2D. Note that the three different regions of interest have the same total area. The left panel shows the input image $I_{0}$. The $\psi D(T_{n}^{m})$ for both input images is the sum $\psi D\left(T_{n}^{m}\right)=\psi D\left(T_{n}^{m}\left(I_{0}\right)\right)+\psi D\left(T_{n}^{m}\left(I_{1}\right)\right)$. The degree of end-stopping of a feature map is then defined as:

(11) $$\phi \left(T_{n}^{m}\right)=1-\frac{1D(T_{n}^{m})}{2D(T_{n}^{m})+\varepsilon }$$

with ε=0.1. Note that the degree of end-stopping is high (close to 1) if the 2D activation is high and the 1D activation is low. However, two special cases were considered: (a) a feature map is “silent,” if all values are very small (i.e., 0D+1D+2D<0.1). (b) The feature map is “0D” if the 0D and 1D activations are similar:

(12) $$T_{n}^{m}\;is\;{}^{'}0D^{'}\Leftrightarrow 1-\frac{0D(T_{n}^{m})}{1D(T_{n}^{m})+\varepsilon }<0.1.$$

For these two special cases, Equation 11 would no longer quantify the degree of end-stopping. Therefore, the degree of end-stopping values was not evaluated for silent and 0D feature maps. The plots in Figure 7 show the normalized histograms for the degree of end-stopping. All bars have a bin width of 0.1 and their heights sum up to 1.

### A.4 Iso-response contours

In this section, we derive the analytical expression for the iso-response contours of FP-neurons. We follow a geometric approach in order to show explicitly how the exo-origin curvature depends on the angle γ=∡(v,g). An alternative approach would be to work with the eigenvector of the symmetric matrix $\frac{1}{2}\left(vg^{T}+gv^{T}\right)$.

In the two-dimensional subspace defined by v and g, and for a specific constant $z\in R^{+}$, we can derive the coordinates of the iso-response contours analytically by using a simplified version of Equation 2: Equation 7. F(x) is the output of the FP-neuron, that is, the product of the outputs of two linear filters v and $g\in R^{n},n=k^{2}$. For simplicity, we disregard the instance normalization. Thus, we assume that the mean values are zero ($\mu_{v}=\mu_{g}=0$) and the standard deviations are 1 ($\sigma_{v}=\sigma_{g}=1$), which are the two variables used for instance normalization. Furthermore, we constrain the input space of x to $S=\{x\in R^{k^{2}}:x^{T}v\geq 0\wedge x^{T}g\geq 0\}$ to account for the ReLU nonlinearities. Furthermore, we restrict γ to [0,π) since for γ=π, both vectors point in opposite directions, and for any point x, one scalar product is always negative.

The optimal stimulus of F(x) is not parallel to one of the filters but points in the direction of the bisector of γ. This property becomes more obvious when rewriting F(x) as a function depending on α=∡(v,x) and β=∡(g,x):

(13) $$F\left(\alpha ,\beta ,x\right)={cos\left(\alpha \right)cos\left(\beta \right)∥v∥∥g∥∥x∥}^{2}.$$

To simplify this particular equation, we assume ∥x∥=1 and disregard the vector lengths ∥v∥ and ∥g∥ since the arguments α and β, and the argmax of F, do not depend on vector length. With α+β=γ, we obtain

(14) $$\begin{array}{ccc} F(\alpha ,\beta ) & \;= & cos(\alpha )cos(\gamma -\alpha )\\ & \;= & \frac{1}{2}\left(cos\left(2\alpha -\gamma \right)+cos\left(\gamma \right)\right).\; \end{array}$$

Note that for $\alpha =\frac{1}{2}\gamma$, F reaches the maximum value $\frac{1+\cos(\gamma )}{2}$.

The subspace of input vectors that do not alter the FP-neuron's output is defined by

(15) $$\begin{array}{c} F\left(x+p\right)=F\left(x\right)\Leftrightarrow p^{T}v=p^{T}g=0.\; \end{array}$$

For any vector p orthogonal to v and g, the iso-response contours are straight, as they are for LN-neurons. However, as we will show in the following, there exists an orthogonal direction o relative to which FP-units exhibit curved iso-response contours and, thus, hyperselectivity.

It is important to note that any input vector x is projected to the plane defined by the vectors v and g (see Equation 7); any vector p from the subspace of Equation 15 is orthogonal to this plane. We can consider the function f(a) that operates on only 2*D* input vectors $a={\left(a,b\right)}^{T}$, which are the projections of x onto the vectors $\frac{v}{∥v∥}$ and $\frac{o}{∥o∥}$, respectively. Unless g is parallel to v, we can derive o as the direction orthogonal to v by using the Gram–Schmidt process:

(16) $$\begin{array}{c} o=g-\frac{v^{T}g}{{∥v∥}^{2}}v.\; \end{array}$$

If g=λv,λ∈R, o is simply any vector orthogonal to v. A point ${\left(a,b\right)}^{T}$ in the two-dimensional projection space can be injected into the original input space S:

(17) $$x_{ab}=\frac{a}{∥v∥}v+\frac{b}{∥o∥}o.$$

$x_{ab}$ denotes that the vector depends on only the position in the projection space $a={\left(a,b\right)}^{T}$. The relations between the scalar products in the input space and the scalar products in the projection space are given by

(18) $$x_{ab}^{T}v=∥v∥\left(a,b\right)e_{1}=a∥v∥$$

(19) $$x_{ab}^{T}o=∥o∥\left(a,b\right)e_{2}=b∥o∥$$

(20) $$\begin{array}{ccc} x_{ab}^{T}g & \;= & {∥g∥\left(a,b\right)\left(cos\left(\gamma \right),sin\left(\gamma \right)\right)}^{T}\\ & \;= & ∥g∥(acos(\gamma )+bsin(\gamma )),\; \end{array}$$

with $e_{1}={\left(1,0\right)}^{T}$ and $e_{2}={\left(0,1\right)}^{T}$. Accordingly, the multiplication of $x^{T}v$ with $x^{T}g$ yields

(21) $$\begin{array}{ccc} x^{T}vx^{T}g & \;= & (a∥v∥)(acos(\gamma )+bsin(\gamma ))∥g∥\\ & \;= & {\left(\begin{array}{c} a^{2}\\ ab \end{array}\right)}^{T}\left(\begin{array}{c} c_{1}cos\left(\gamma \right)\\ c_{1}sin\left(\gamma \right) \end{array}\right)=f\left(a\right),\; \end{array}$$

with $c_{1}=∥v∥∥g∥$. In the projection space, the direction vector of the optimal stimulus $a_{opt}$ is given by ${\left(cos\left(\frac{\gamma }{2}\right),\sin\left(\frac{\gamma }{2}\right)\right)}^{T}$ (see Equation 14). $a_{orth}={\left(-sin\left(\frac{\gamma }{2}\right),\cos\left(\frac{\gamma }{2}\right)\right)}^{T}$ is orthogonal to it. We aim to find all points x,y∈R such that

(22) $$f(xa_{orth}+ya_{opt})=z,$$

with $z\in R^{+}$. Substitution and simplification yields:

(23) $$\begin{array}{c} z=c_{1}\left(y^{2}cos^{2}\left(\frac{\gamma }{2}\right)-x^{2}sin^{2}\left(\frac{\gamma }{2}\right)\right).\; \end{array}$$

For a given value x, and $c=\frac{c_{1}}{z}$, the y position of the iso-response contour is given by

(24) $$\begin{array}{c} y\left(x\right)=\sqrt{tan^{2}\left(\frac{\gamma }{2}\right)x^{2}+\frac{c}{cos^{2}\left(\frac{\gamma }{2}\right)}}.\; \end{array}$$

With this equation, we can estimate the curvature of the exo-origin bend by using the quadratic coefficient of the second-order Taylor approximation around x=0 to obtain

(25) $$\begin{array}{c} \frac{1}{2}\left[\frac{d^{2}}{dx^{2}}\left(y\right)\right]\left(0\right)=\frac{tan^{2}\left(\frac{\gamma }{2}\right)}{2\sqrt{\frac{c}{cos^{2}\left(\frac{\gamma }{2}\right)}}}.\; \end{array}$$

For x=0, y(0) is the position along the optimal stimulus, where $f(y\left(0\right)a_{opt})=z$. Keeping y(0) fixed, the attenuation of f when moving in a direction orthogonal to the optimal stimulus is quadratic:

(26) $$\begin{array}{ccc} \Delta z & \;= & f\left(xa_{orth}+y\left(0\right)a_{opt}\right)-f\left(y\left(0\right)a_{opt}\right)\\ & \;= & -c_{1}x^{2}sin^{2}\left(\frac{\gamma }{2}\right).\; \end{array}$$

Figure 14 gives a three-dimensional example to illustrate how a 3*D* point $p\in R^{3}$ can be mapped to the plane spanned by v and g. The axes a and b of the projection space coincide with v and o. Thus, there is a direct correspondence between p and the projected point $q={\left(a,b\right)}^{T}$ (see Equation 17). To estimate the curvature, we rotate the (a,b) coordinate frame clockwise by $\frac{\pi -\gamma }{2}$ to the frame (x,y). From this perspective, we can measure the change of y when moving along the x-axis and away from x=0: Equation 24 shows that for γ∈(0,π), y(x) increases when changing x. Accordingly, the iso-response contour bends away from the origin of the rotated frame (x,y).

### A.5 Adversarial attacks

The mean robust errors (i.e., the errors regarding the perturbed images), in percentages, averaged over five runs for different architectures and ε-values are given in Table 2, and the averaged percentages of changed predictions (see Equation 9) are given in Table 3. We show a selection of adversarial examples in Figure 15. We observed that, for the basic block networks, the FP-net (basic) consistently outperformed the ResNet for all Ns except N=3. For the pyramid block networks, the larger FP-nets (N∈{7,9}) consistently outperformed the PyrBlockNets. Accordingly, especially for larger CNNs, we increased the robustness against adversarial attacks by using FP-blocks. To compute the FGSM attacks for each test image, we used the code provided by Kim (2020). The RGB test images were first divided by 255, then the FGSM algorithm was applied, and finally, the image was normalized as described above.

**Table 2.** Robust error values, in percentages, when using FGSM perturbations for all Cifar-10 models and different ε values. We report the mean values averaged over five different training runs.

Model; ε=	1/255	2/255	4/255	8/255	16/255
FP-net (N=3)	58.460	72.766	82.036	86.180	86.444
FP-net (basic) (N=3)	56.662	73.634	82.296	86.406	89.108
PyrBlockNet (N=3)	60.458	75.566	83.992	88.150	89.762
ResNet (N=3)	57.284	71.912	80.378	84.338	88.016
FP-net (N=5)	53.634	70.694	81.088	85.020	88.152
FP-net (basic) (N=5)	51.112	68.510	79.036	84.034	86.830
PyrBlockNet (N=5)	54.620	70.018	79.748	84.392	87.666
ResNet (N=5)	52.434	69.652	79.678	84.166	86.956
FP-net (N=7)	51.082	67.468	78.728	83.510	86.814
FP-net (basic) (N=7)	48.360	66.104	77.236	82.486	86.256
PyrBlockNet (N=7)	53.244	69.966	80.764	85.880	88.662
ResNet (N=7)	49.788	67.852	78.590	83.290	86.544
FP-net (N=9)	49.226	66.902	78.634	83.524	87.216
FP-net (basic) (N=9)	45.896	64.284	76.076	81.652	85.484
PyrBlockNet (N=9)	52.048	68.442	78.932	84.590	87.620
ResNet (N=9)	47.888	66.056	77.066	81.992	86.410

**Table 3.** Percentage of changed predictions when using the FGSM perturbations for all Cifar-10 models and different ε values.

Model; ε=	1/255	2/255	4/255	8/255	16/255
FP-net (*N =* 3)	50.766	65.140	74.596	79.320	82.030
FP-net (basic) (*N =* 3)	48.858	65.928	74.906	80.128	85.654
PyrBlockNet (*N =* 3)	52.560	67.818	76.752	82.592	87.562
ResNet (*N =* 3)	49.526	64.228	73.038	78.480	85.148
FP-net (*N =* 5)	47.030	64.132	74.858	80.258	86.418
FP-net (basic) (*N =* 5)	44.482	61.960	72.662	78.526	83.700
PyrBlockNet (*N =* 5)	47.808	63.416	73.736	80.064	85.782
ResNet (*N =* 5)	44.996	62.298	72.546	78.148	83.916
FP-net (*N =* 7)	44.968	61.452	73.000	78.932	84.772
FP-net (basic) (*N =* 7)	42.102	59.922	71.294	77.402	83.376
PyrBlockNet (*N =* 7)	47.054	63.978	75.342	81.884	86.844
ResNet (*N =* 7)	42.820	60.948	71.898	77.538	83.390
FP-net (*N =* 9)	43.536	61.314	73.444	79.506	85.424
FP-net (basic) (*N =* 9)	39.804	58.240	70.196	76.448	82.376
PyrBlockNet (*N =* 9)	45.988	62.592	73.590	80.620	85.988
ResNet (*N =* 9)	41.206	59.426	70.662	76.534	83.808

### A.6 JPEG-compression

The mean robust errors percentages averaged over five runs are given in Table 4, and the averaged percentages of changed predictions are given in Table 5. Examples for JPEG-compressed images depending on the quality level are given in Figure 16. To compute the compression, we used the software provided by the OpenCV library (Bradski, 2000). We made the following observations: A decrease in quality by 10 was followed by an error increase of roughly 3–4%. Analogously, each quality decrease increased the number of changed predictions by 3–4%. Deeper networks performed better. Networks using the basic block, the ResNet and the FP-net (basic), performed better than networks based on the pyramid block. Except for a quality of 10, the FP-net (basic) outperformed the ResNet, and similarly, the FP-net outperformed the PyrBlockNet. From this, we concluded that using FP-blocks in a CNN increased the robustness against noise coming from JPEG-compression.

**Table 4.** Error values in percentages when using JPEG-compression for all Cifar-10 models and different quality (Q) values.

Model; Q=	90	80	70	60	50	40	30	20	10
FP-net (*N =* 3)	12.412	16.468	19.686	22.624	25.230	28.116	32.920	41.044	57.940
FP-net (basic) (*N =* 3)	12.288	16.338	19.636	22.734	25.306	27.890	32.744	40.138	56.260
PyrBlockNet (*N =* 3)	13.080	17.356	20.676	23.790	27.108	30.324	35.326	42.798	57.336
ResNet (*N =* 3)	12.910	17.322	20.830	24.416	27.308	30.404	35.252	43.356	58.722
FP-net (*N =* 5)	11.040	15.154	18.742	22.076	24.676	27.864	32.772	41.240	58.466
FP-net (basic) (*N =* 5)	10.908	14.866	18.142	21.282	23.792	26.760	31.736	40.024	58.488
PyrBlockNet (*N =* 5)	11.918	16.412	20.342	23.898	27.078	30.694	35.888	44.424	60.176
ResNet (*N =* 5)	11.592	15.654	19.114	22.330	24.590	27.462	32.100	40.726	56.878
FP-net (*N =* 7)	10.456	14.596	18.214	21.292	24.112	26.720	31.768	40.582	58.022
FP-net (basic) (*N =* 7)	10.624	14.274	17.590	20.384	23.020	26.014	30.646	39.382	56.966
PyrBlockNet (*N =* 7)	11.396	15.928	19.344	23.356	26.518	30.456	35.908	44.706	59.660
ResNet (*N =* 7)	11.338	15.294	18.288	21.254	23.968	27.044	31.534	40.168	56.540
FP-net (*N =* 9)	10.118	14.228	18.020	21.116	23.820	26.894	32.382	40.830	57.930
FP-net (basic) (*N =* 9)	10.128	13.984	16.996	19.912	22.116	24.956	29.710	38.096	56.040
PyrBlockNet (*N =* 9)	10.974	15.290	19.088	22.640	25.504	29.244	34.520	43.218	59.616
ResNet (*N =* 9)	10.788	14.830	18.162	21.558	24.200	27.728	32.272	40.942	57.842

**Table 5.** Percentage of changed predictions when using JPEG-compression for all Cifar-10 models and different quality (Q) values.

Model; Q=	90	80	70	60	50	40	30	20	10
FP-net (*N =* 3)	9.024	14.124	17.676	21.014	23.714	26.688	31.580	40.116	57.502
FP-net (basic) (*N =* 3)	8.868	13.914	17.602	21.064	23.636	26.590	31.540	39.208	55.918
PyrBlockNet (*N =* 3)	10.112	15.086	18.864	22.176	25.670	29.102	34.168	41.842	56.930
ResNet (*N =* 3)	9.996	15.192	19.108	22.826	25.972	29.366	34.340	42.522	58.384
FP-net (*N =* 5)	8.374	13.158	17.104	20.644	23.232	26.690	31.766	40.412	58.090
FP-net (basic) (*N =* 5)	8.146	12.820	16.488	19.986	22.540	25.586	30.744	39.296	58.002
PyrBlockNet (*N =* 5)	9.322	14.590	18.746	22.488	25.770	29.656	35.006	43.668	59.840
ResNet (*N =* 5)	8.650	13.584	17.396	20.806	23.168	26.280	31.188	39.802	56.370
FP-net (*N =* 7)	8.058	12.868	16.780	19.898	22.830	25.714	30.818	39.848	57.782
FP-net (basic) (*N =* 7)	7.832	12.222	15.932	18.960	21.662	24.748	29.682	38.688	56.742
PyrBlockNet (*N =* 7)	8.804	13.980	17.920	22.114	25.410	29.432	34.964	44.002	59.232
ResNet (*N =* 7)	8.478	13.066	16.464	19.640	22.540	25.884	30.492	39.142	55.986
FP-net (*N =* 9)	7.694	12.482	16.704	19.866	22.700	25.886	31.402	40.064	57.774
FP-net (basic) (*N =* 9)	7.632	12.124	15.522	18.564	21.014	23.992	28.952	37.426	55.786
PyrBlockNet (*N =* 9)	8.740	13.498	17.594	21.258	24.294	28.190	33.596	42.560	59.332
ResNet (*N =* 9)	8.180	12.760	16.728	20.056	22.892	26.606	31.298	40.196	57.468

## References

[bib1] Barlow, H. (1961). Possible principles underlying the transformation of sensory messages. *Sensory Communication,* 1(1), 217–234.

[bib2] Barth, E., & Watson, A. B. (2000). A geometric framework for nonlinear visual coding. *Optics Express,* 7(4), 155–165. Available from http://webmail.inb.uni-luebeck.de/inb-publications/pdfs/BaWa00.pdf. 19407860 10.1364/oe.7.000155

[bib3] Barth, E., & Zetzsche, C. (1998). Endstopped operators based on iterated nonlinear center-surround inhibition. In B. E. Rogowitz & T. N. Pappas (Eds.), *Human vision and electronic imaging* (Vol. 3299, pp. 67–78). Bellingham, WA: Optical Society of America, Available from http://webmail.inb.uni-luebeck.de/~barth/papers/spie98.fm4.pdf.

[bib4] Bradski, G. (2000). The openCV library. *Dr. Dobb's Journal: Software Tools for the Professional Programmer,* 25(11), 120–123.

[bib5] Chrysos, G., et al. (2020). P-nets: Deep polynomial neural networks. In *2020 IEEE/CVF Conference on Computer Vision and Pattern Recognition (CVPR)*, Seattle, WA, USA, pp. 7323–7333, doi:10.1109/CVPR42600.2020.00735.

[bib6] Collins, J., Sohl-Dickstein, J., & Sussillo, D. (2016). Capacity and trainability in recurrent neural networks. *Stat,* 1050, 29.

[bib7] Deng, J., Dong, W., Socher, R., Li, L.-J., Li, K., & Fei-Fei, L. (2009). ImageNet: A large-scale hierarchical image database. *2009 IEEE Conference on Computer Vision and Pattern Recognition*, pp. 248–255, doi:10.1109/CVPR.2009.5206848.

[bib8] Goodfellow, I. J., Shlens, J., & Szegedy, C. (2015). Explaining and harnessing adversarial examples. In Bengio, Y., LeCun, Y. (Eds.), *3rd International Conference on Learning Representations, ICLR 2015, San Diego, CA, USA, Conference Track Proceedings*. Opgehaal van, http://arxiv.org/abs/1412.6572.

[bib9] Gray, G. (2017). *Sequential**-**imagenet**-**dataloader*. Retrieved February 20, 2021, from, https://github.com/BayesWatch/sequential-imagenet-dataloader.

[bib10] Grüning, P., & Barth, E. (2021a). Bio-inspired min-nets improve the performance and robustness of deep networks. In *SVRHM 2021 Workshop @ NeurIPS*, https://openreview.net/forum?id=zxxdFLB8F24.

[bib11] Grüning, P., & Barth, E. (2021b). Fp-nets for blind image quality assessment. *Journal of Perceptual Imaging,* 4(1), 10402-1–10402-13.

[bib12] Grüning, P., Martinetz, T., & Barth, E. (2020a). Feature products yield efficient networks. *arXiv* *preprint arXiv:2008.07930*.

[bib13] Grüning, P., Martinetz, T., & Barth, E. (2020b). Log-nets: Logarithmic feature-product layers yield more compact networks. In I. Farkaš, P. Masulli, & S. Wermter (Eds.), *Artificial Neural Networks and Machine Learning – ICANN 2020* (pp. 79–91). Cham, Switzerland: Springer International Publishing.

[bib14] Han, D., Kim, J., & Kim, J. (2017). Deep pyramidal residual networks. In *2017 IEEE Conference on Computer Vision and Pattern Recognition (CVPR)*, Honolulu, HI, USA, pp. 6307–6315, doi:10.1109/CVPR.2017.668.

[bib15] He, K., Zhang, X., Ren, S. & Sun, J. (2016). Deep residual learning for image recognition. *2016 IEEE Conference on Computer Vision and Pattern Recognition (CVPR)*, pp. 770–778, doi:10.1109/CVPR.2016.90.

[bib16] Howard, J. (2018). *Imagenet**-fast**.* https://github.com/fastai/imagenet-fast. Accessed February 20, 2021.

[bib17] Hubel, D. H., & Wiesel, T. N. (1965). Receptive fields and functional architecture in two nonstriate visual areas (18 and 19) of the cat. *Journal of Neurophysiology,* 28(2), 229–289. 14283058 10.1152/jn.1965.28.2.229

[bib18] Kim, H. (2020). Torchattacks: A pytorch repository for adversarial attacks. *arXiv* *preprint arXiv:**2010.01950*.

[bib19] Krizhevsky, A., Nair, V., & Hinton, G. (2021). *Cifar-10 (**Canadian Institute for Advanced Research).*

[bib20] LeCun, Y., Bengio, Y., & Hinton, G. (2015). Deep learning. *Nature,* 521(7553), 436–444. 26017442 10.1038/nature14539

[bib21] Li, D., Zhou, A., & Yao, A. (2021). *Mobilenetv2**.pytorch.* Retrieved February 20, 2021, from https://github.com/d-li14/mobilenetv2.pytorch.

[bib22] Li, Y., Wang, N., Liu, J., & Hou, X. (2017). Factorized bilinear models for image recognition. In *2017 IEEE International Conference on Computer Vision (ICCV), Venice, Italy*, pp. 2098–2106, doi:10.1109/ICCV.2017.229.

[bib23] Lu, L. (2020). Dying ReLU and initialization: Theory and numerical examples. *Communications in Computational Physics,* 28(5), 1671–1706, doi:10.4208/cicp.OA-2020-0165.

[bib24] Majaj, N. J., & Pelli, D. G. (2018). Deep learning—Using machine learning to study biological vision. *Journal of Vision,* 18(13), 2–2. 10.1167/18.13.2 PMC6279369 30508427

[bib25] Mel, B. W., & Koch, C. (1990). Sigma-pi learning: On radial basis functions and cortical associative learning. In Touretzky, D. (Ed.), *Advances in Neural Information Processing Systems,* 2.

[bib26] Mota, C., & Barth, E. (2000). On the uniqueness of curvature features. *Dynamische Perzeption,* 9, 175–178, Available from https://webmail.inb.uni-luebeck.de/inb-publications/htmls/ulm2000.html.

[bib27] Paiton, D. M., Frye, C. G., Lundquist, S. Y., Bowen, J. D., Zarcone, R., & Olshausen, B. A. (2020). Selectivity and robustness of sparse coding networks. *Journal of Vision,* 20(12), 10, 10.1167/jov.20.12.10. PMC7691792 33237290

[bib28] Paszke, A., Gross, S., Massa, F., Lerer, A., Bradbury, J., Chanan, G. et al. (2019). Pytorch: An imperative style, high-performance deep learning library. In Wallach, H., Larochelle, H., Beygelzimer, A., d'Alché-Buc, F., Fox, E., Garnett, R., Paszke, A., Gross, S., Massa, F., Lerer, A., Bradbury, J., Chanan, G., Chintala, S. (Eds.), *Advances in neural information processing systems* (Vol. 32, pp. 8026–8037). Red Hook, NY: Curran Associates, Inc.

[bib29] Rao, R. P., & Ballard, D. H. (1999). Predictive coding in the visual cortex: A functional interpretation of some extra-classical receptivefield effects. *Nature Neuroscience,* 2(1), 79–87. 10195184 10.1038/4580

[bib30] Rumelhart, D. E., Hinton, G. E., & McClelland, J. L. (1986). A general framework for parallel distributed processing. *Parallel Distributed Processing: Explorations in the Microstructure of Cognition,* 1(26), 45–76.

[bib31] Sandler, M., Howard, A., Zhu, M., Zhmoginov, A., & Chen, L. (2018). MobileNetV2: Inverted residuals and linear bottlenecks. In *2018 IEEE/CVF Conference on Computer Vision and Pattern Recognition (CVPR)*, Salt Lake City, UT, USA, pp. 4510–4520, doi:10.1109/CVPR.2018.00474.

[bib32] Simoncelli, E. P., & Olshausen, B. A. (2001). Natural image statistics and neural representation. *Annual Review of Neuroscience,* 24(1), 1193–1216. 10.1146/annurev.neuro.24.1.1193 11520932

[bib33] Srivastava, R. K., Greff, K., & Schmidhuber, J. (2015). Training very deep networks. *Proceedings of the 28th International Conference on Neural Information Processing Systems - Volume 2*, pp. 2377–2385. Presented at the Montreal, Canada. Cambridge, MA, USA: MIT Press.

[bib34] Szegedy, C., Liu, W., Jia, Y., Sermanet, P., Reed, S., Anguelov, D. et al. (2014). *Going deeper with convolutions.*

[bib35] Szegedy, C., Zaremba, W., Sutskever, I., Bruna, J., Erhan, D., Goodfellow, I., & Fergus, R. (2014). *Intriguing properties of neural networks.* Paper presented at 2nd International Conference on Learning Representations, ICLR 2014, Banff, Canada.

[bib36] Ulyanov, D., Vedaldi, A., & Lempitsky, V. (2016). Instance normalization: The missing ingredient for fast stylization. *arXiv* *preprint arXiv:1607.**08022*.

[bib37] Veit, A., Wilber, M. J., & Belongie, S. (2016). Residual networks behave like ensembles of relatively shallow networks. In Lee, D., Sugiyama, M., Luxburg, U., Guyon, I., Garnett, R. (Eds.), *Advances in Neural Information Processing Systems* (Vol 29). Curran Associates, Inc.

[bib38] Vilankar, K. P., & Field, D. J. (2017). Selectivity, hyperselectivity, and the tuning of v1 neurons. *Journal of Vision,* 17(9), 9, 10.1167/17.9.9. 28813565

[bib39] Watanabe, S. (1985). *Pattern Recognition: Human and Mechanical*. Hoboken, New Jersey: Wiley-Interscience.

[bib40] Wu, Y. (2016). *Tensorpack*, https://github.com/tensorpack/tensorpack/tree/master/examples/ResNet. Accessed February 20, 2021.

[bib41] Zetzsche, C., & Barth, E. (1990). Fundamental limits of linear filters in the visual processing of two-dimensional signals. *Vision Research,* 30, 1111–1117. Available from http://webmail.inb.uni-luebeck.de/inb-publications/pdfs/ZeBa90a.pdf. 2392840 10.1016/0042-6989(90)90120-a

[bib42] Zetzsche, C., Barth, E., & Wegmann, B. (1993). The importance of intrinsically two-dimensional image features in biological vision and picture coding. In A. B. Watson (Ed.), *Digital images and human vision* (pp. 109–38). Cambridge, MA: MIT Press. Available from http://webmail.inb.uni-luebeck.de/inb-publications/htmls/ZeBaWe93a.html.

[bib43] Zoumpourlis, G., Doumanoglou, A., Vretos, N., Daras, P. Non-linear convolution filters for CNN-based learning. In *2017 IEEE International Conference on Computer Vision (ICCV)*, Venice, Italy, pp. 4771–4779, doi:10.1109/ICCV.2017.510.

